# Adjuvants in IVF—evidence for what works and what does not work

**DOI:** 10.1080/03009734.2020.1751751

**Published:** 2020-05-07

**Authors:** Luciano Nardo, Spyridon Chouliaras

**Affiliations:** a Reproductive Health Group, Daresbury Park, UK;; b Manchester Metropolitan University, Manchester, UK;; c Sidra Medicine, Doha, Qatar;; d Weill Cornell Medicine, Ar-Rayyan, Qatar

**Keywords:** Adjuvant therapy, IVF

## Abstract

The field of assisted reproductive technology is shaped and changed constantly by advances in science and cutting-edge innovations. In a quest to maximise outcomes, add-on interventions are often adopted and utilised prematurely while the principles of evidence-based medicine seem to be less strictly adhered to. In this review we will attempt to summarise the latest evidence about some of the adjuvants.

## Introduction

Known also as treatment add-ons, adjuvants or adjuncts are additional, optional, treatment steps that may be offered on top of standard fertility treatments. The topic of add-ons has been heavily debated as many consider such treatments unfounded.

However, one has to agree that the very reason that this kind of treatments exists, and new innovative treatments keep emerging, is the challenge faced by the clinicians along with the frustration experienced by those trying to conceive.

When clinics start offering such treatments a Pandora’s box opens, as the question then would be where one draws the line. Worldwide the *in vitro* fertilisation (IVF) industry is already worth billions of dollars and is booming so there seems to be no easy answer to this question.

As the debate regarding the provision of add-ons is ongoing, several regulatory bodies and professional learned societies have released guidance and statements urging the public to be cautious and to question the use of such treatment modalities. Among those bodies are the Royal College of Obstetricians and Gynaecologists (RCOG) and Human Fertilisation and Embryology Authority (HFEA) in the UK, and the Australian-based Victorian Assisted Reproductive Treatment Authority (VARTA). The issue of add-ons has been under the spotlight even by investigative undercover journalists who have condemned the practice of some private clinics.

Large randomised trials in IVF are very hard to pursue as patients are very keen to try novel or already established empirical therapies and therefore being randomised to a placebo arm is unappealing for them. For most of the add-ons presented in this publication, the available evidence is suboptimal, and better-designed studies are essential to give conclusive answers. We will therefore refrain from repeating the same argument separately for each treatment modality. We will also not elaborate on strategies used in order to improve success rates such as: freeze-all, routine use of ICSI for non-male factor infertility, preimplantation genetic testing for all cycles, continuous monitoring of the embryos (time lapse imaging), testing for endometrial receptivity or endometrial microbiome, and advanced sperm selection techniques such as intracytoplasmic morphologically selected sperm injection (IMSI), physiological ICSI (PICSI), and sperm head’s birefringence. It is our belief that these do not strictly speaking constitute adjuvants to treatment, and the decision regarding their use may be influenced by other factors. Instead we will try to focus on what we consider as ‘pure’ add-ons to treatment.

## IVF laboratory-related adjuvants

### Embryo glue and adherence compounds

The idea of a using a substance that could facilitate blastocyst implantation originates in the early days of IVF. Fibrin sealants have failed to demonstrate significant improvement in clinical outcomes; however, lately the spotlight has been on a specific culture medium with added hyaluronan, marketed as EmbryoGlue. Hyaluronan is a glycoprotein which is well known to provide a high viscosity environment in the uterus. It has also been observed that the synthesis of intrauterine hyaluronan increases before implantation and goes back to near basal levels after. The latest Cochrane review of 3898 participants from 17 randomised control trials (RCTs) demonstrated moderate-quality evidence for an improvement in clinical pregnancy rates (CPR) and live birth rates (LBR), with an associated increase in multiple pregnancy rate, when transfer medium was supplemented with hyaluronan. The published evidence may be suggestive of a beneficial effect of the use of hyaluronan-supplemented embryo transfer media (1).

### Assisted hatching

Assisted hatching, which is effectively the artificial disruption of the zona pellucida, has been used since the early years of assisted reproductive technology (ART), but its popularity is fading. Chemical, mechanical, laser-assisted hatching, or even drilling of the zona could in theory aid a hardened zona to break and thus facilitate the transfer of metabolites, growth factors, and signals between the embryo and the endometrium.

Reviews on assisted hatching have reported statistically significant increases in CPR but no evidence for a difference in LBR. The latest meta-analysis of studies on assisted hatching included 36 RCTs with 6459 participants and found that assisted hatching gave a significant increase in CPR, but in the 15 RCTs that looked at LBR there was no evidence of benefit from assisted hatching (2).

### Artificial oocyte activation (AOA)

Failure of oocyte activation is thought to be an important cause of fertilisation failure following conventional ICSI. Artificially inducing oocyte activation by the use of calcium ionophores has been trialled for women with previous low or total failed fertilisation in previous ICSI cycle(s). The suggested physiological mechanism of action is by increasing calcium ion concentration around the ooplasm immediately following sperm–oocyte fusion, although oocyte activation is very complex and therefore the understanding is not complete.

The most widespread AOA agents used in human ART are two Ca2þ-selective ionophores—ionomycin and A23187 (also known as calcimycin)—although a protocol using strontium chloride (SrCl_2_) has also been reported.

There have been several promising reports regarding application of this technology; however, there is significant heterogeneity in the AOA protocols as well as the indications for its use. A systematic review and meta-analysis of RCTs concluded that there was insufficient evidence to prove the effectiveness of AOA. The data were considered dissimilar and of low quality, with studies having various risks of bias (3).

Data regarding the safety of this intervention have been reassuring till now, and its use in the context of scientific trials can be justified.

## Patient-centered adjuvants

### Improvement of blood supply

*Heparin*. Heparin offers prophylaxis against formation of micro-thrombi at the implantation site by inhibiting clotting factor Xa. Both unfractionated and low-molecular-weight heparin (LMWH) have been used to promote successful invasion of trophoblasts in the presence of antiphospholipid syndrome. Low-molecular-weight heparin’s favourable pharmacological profile is the obvious reason why it is almost exclusively used in the context of ART, and therefore we will mostly focus on evidence regarding LMWH. The role of heparin in women suffering from thrombophilia who are undergoing assisted conception treatment is established, but its use in the general IVF population is still a grey area. One meta-analysis that included RCTs of women with repeated implantation failure (RIF) after ≥3 failed embryo transfer cycles (4) and a Cochrane review that included a RCT of women undergoing their first IVF cycle (5) found improvement in LBR (77–79%) with the use of LMWH. However, they both concluded that its routine use cannot be advised as the evidence is seriously limited by inconsistency, imprecision, and potentially the small sample and statistical methodology. In a third meta-analysis authors gave a similar message. They demonstrated significantly higher CPR and LBR in the observational studies but not when sensitivity analyses included women with RIF. When the RCTs’ data were pooled there was again no evidence of benefit (6). A more recent large retrospective study in women with two or more unsuccessful IVF cycles failed to demonstrate any benefit from the addition of LMWH (7). There is growing evidence that, other than the antithrombotic effect, LMWH may favour reproductive outcomes via different mechanisms of action, such as modulating the decidualization of endometrial stromal cells and thus endometrial receptivity (8) or increasing heparin-binding epidermal growth factor (HBEGF) expression, which in turn is required to stimulate early trophoblast differentiation and invasion (9). These anti-thrombin-independent effects of LMWH suggest that we should look at the evidence of heparin use in ART with a fresh eye, and therefore larger studies with a different design are desired. Since the use of LMWH is not without risk, like bleeding and thrombocytopenia, the use at the moment in the general IVF population or even in women with implantation failure requires appropriate counselling in light of the limitations of available evidence.

*Aspirin*. Acetylsalicylic acid is one of the most commonly used medications worldwide and usually prescribed for the prevention of cardiovascular disease due to its role as an antiplatelet-aggregation agent. Low-dose aspirin also results in vasodilation and increase in the peripheral blood flow by inducing a shift from thromboxane A2 to prostacyclin. Evidence on the prophylactic role of aspirin against pre-eclampsia and intrauterine growth restriction from the CLASP study resulted in its use being adopted for treatment of embryo implantation failure. Moreover, it has been shown that aspirin improves uterine and ovarian arteries doppler indices, which in turn leads to improved oocyte quality and favours implantation rates (10). A meta-analysis failed to support its routine use for women undergoing IVF (11). Similarly, a Cochrane review of 13 RCTs (12) suggested that administration of aspirin did not improve pregnancy rates for IVF patients. More recently, another Cochrane review of studies in women with recurrent miscarriage, whether associated with thrombophilia or not, did not find any benefit of the use of aspirin alone or in combination with LMWH when trials with high risk of bias were excluded (13). Therefore, routine aspirin use is not advocated.

*Sildenafil*. Sildenafil citrate acts as a vasodilator by potentiating the effect of nitric oxide on vascular smooth muscle. It was therefore speculated that improving the blood supply and oxygenation of the uterus could enhance implantation rates. Vaginal sildenafil citrate (Viagra) is predominantly being used for treating cases with thin endometrium. A retrospective study suggested enhancement of endometrial development in 70% of patients, while high implantation and pregnancy rates were achieved in a cohort of poor-prognosis patients (14). In contrast, another study, where sildenafil supplementation was given in women undergoing fresh IVF or frozen embryo transfer, failed to demonstrate an increase in endometrial thickness or blood supply (15). In general, studies which examine sildenafil’s use are underpowered, heterogeneous, and often its use is combined with other medications. Its routine use cannot be recommended outside a research setting.

### Antioxidants

In humans, excess formation of reactive oxygen species (ROS) can induce oxidative stress and lead to cell damage or death. Cells have antioxidant mechanisms which regulate this process in order to achieve homeostasis, but often it becomes unbalanced. High levels of free radicals are thought to affect the reproductive function; therefore, antioxidants are increasingly being used over the counter by patients but also recommended by clinicians to improve natural reproductive potential and IVF outcomes.

In male infertility their role is relatively well documented, and recently the term male oxidative stress infertility (MOSI) has been suggested as a distinct category (16). However, supplementation with antioxidant in women has not always demonstrated similar results (17).

The presence of a dominant Th1 phenotype is a common finding in women with recurrent miscarriages and implantation failure. An antioxidant diet regime has been shown to improve the cytokine ratio and therefore may indirectly lead to improved reproductive outcomes (18). One of the antioxidants, coenzyme Q10 (CoQ-10), which is an essential component of the inner mitochondrial membrane, has particularly been of interest due to its effect in the energy production of the oocyte.

Mitochondrial dysfunction is implicated with ovarian aging in both animal and human models. Preliminary work suggested that supplementation with CoQ-10 may reverse this process (19). It has even been shown that CoQ-10 improves oocyte and cumulus cells quantity and quality (20), while a recent RCT has suggested improvement in ovarian response and embryo quality but due to sample size failed to demonstrate improvement in clinical outcomes (21). Still, the optimal duration and dosage of CoQ-10 supplementation remain elusive.

Finally, it should be stressed that some ROS are signalling molecules in cellular signalling pathways, and therefore lowering their numbers may not be beneficial. It seems that both extremes—oxidative and antioxidative stress—are undesired, and blindly prescribing antioxidant supplements without an accurate determination of the oxidative stress may be too simplistic and counterproductive (22).

### Immunotherapy

There is no doubt that the immune regulation of the dialogue between the endometrium and the embryo during implantation is fundamental. The so-called immunological paradox of pregnancy though is still not well understood (23). Women desperate to find an explanation for their repeated IVF failures are often worried that their bodies may be rejecting embryos or that the gametes could be incompatible. The anxiety surrounding these theories is only enhanced by unfortunate and sometimes irresponsible media publications and by the specific terminology used in reproductive immunology (e.g., killer, necrosis, etc.). Natural killer (NK) cells, cytokines, tumour necrosis factor-alpha (TNF-α), and the balance between T-helper cells (Th1/Th2) all play a major role in the embryo implantation process.

Tests used to identify irregularities in the regulation of reproductive immunology such as peripheral blood NK (pbNK) cell number and activity and uterine NK (uNK) cell number and activity are gaining popularity. The relevance of some of these tests is questionable as there is a lack of consensus on the methods used for measuring and reporting as well as lack of a clear definition of a ‘normal’ range or what constitutes a ‘high’ cell count for either pbNK or uNK cells (24).

A systematic review found no significant difference in the pbNK cell number and activity in women with or without implantation failure or miscarriage after ART. The authors concluded that the prognostic value of routine testing for pbNK cells is unclear. The implantation rates in women with or without elevated uNK cells in the two studies included in this review were comparable (25). Below we will attempt to further elaborate on available evidence—or lack thereof—for the most commonly prescribed immune treatments.

#### Steroids

Different regimes of oral prednisolone or dexamethasone are being prescribed as an adjuvant to IVF. It has been proposed that glucocorticoids may improve the intrauterine environment due to the anti-inflammatory and immune-suppressive activity by acting as immunomodulators to normalise the cytokine expression profile and by reducing the uNK cell count and activity in the endometrium, thus suppressing endometrial inflammation (26). A Cochrane review of 14 RCTs found no benefit of glucocorticoids on LBR, CPR, and miscarriage rates when used in an unselected IVF/ICSI population, except in a subgroup of IVF rather than ICSI cycles (OR 1.5, 95% CI 1.05–2.13), which is biologically difficult to interpret (27).

There have been several studies though looking into more specific groups of women having IVF and treated with steroids alone or combined with other medications, like women with RIF or recurrent miscarriages or even women with abnormal immune testing such as elevated NK cells, or the presence of anti-cardiolipin antibodies, thyroid peroxidase antibodies, and anti-nuclear antibodies. One meta-analysis on the use of corticosteroids in women undergoing IVF with elevated pbNK cells found only one study which demonstrated an increase in CPR with prednisolone (OR 1.63, 95% CI 1.00–2.66). The quality of evidence was considered low to indicate improved IVF outcomes (28). Another meta-analysis found significant improvement in LBR and reduction of miscarriage rates when women with idiopathic recurrent miscarriages were treated with prednisolone (29). Prednisolone used as an adjunct along LMWH in women with one or more previous unexplained failed IVF or ICSI cycles has also been studied. Both studies suggested an improvement in IVF or ICSI outcomes associated with adjuvant therapy (30,31). The interpretation of all the available evidence is complex, but there may be some rationale for the use of oral steroids in women with RIF or recurrent miscarriages with an abnormal immunological profile. Due to the low cost and perceived safety in short-term use, except from a weak association with cleft palate (32), prednisolone may be offered in selected cases unless future evidence directs otherwise.

#### Intralipids

Infusion of intralipids is used for parenteral nutrition and is a fatty emulsion containing soya bean oil, egg yolk, phospholipids, glycerine, and water. It is also used in emergencies to treat systemic toxicity induced by local anaesthetics. Increasingly it is being used for women with failed IVF attempts and abnormal NK cell activity (33).

A double-blind randomised control trial of 296 women with secondary infertility, recurrent miscarriage, and elevated pbNK cells showed no increase in CPR in the group of women who were treated with intralipid infusion (34). Another prospective trial in a very selected cohort of patients had to be terminated early as the results favoured the group which did not receive intralipids therapy (35).

A retrospective study of women who received intralipids infusion due to elevated pbNK cells failed to identify any benefit, and a cost-effectiveness analysis deemed that the treatment was not cost-effective (36).

Therefore, despite the relatively low cost of the intervention and the good safety profile, the evidence for its use is lacking, and prospective patients should be made aware of this.

#### Immunoglobulin

Theories regarding the potential mechanisms of actions of i.v. immunoglobulin (IVIg) include inhibition of pbNK cell numbers and/or activity, correction of abnormal Th1/Th2 ratio, but also non-specific immunomodulation leading to enhancement of immunological tolerance. A systematic review published in 2013 included 10 studies and a total of 835 women who received IVIg as adjuvant treatment to IVF. A significantly higher implantation rate (RR 2.708, 95% CI 1.302–5.629), CPR (RR 1.463, 95% CI 1.075–1.991), and LBR (RR 1.616, 95% CI 1.243–2.101) were reported, as well as a significantly lower miscarriage rate (RR 0.352, 95% CI 0.168–0.738). Although this review showed that IVIg use was favourable for all reproductive outcomes, the quality of the retrospective studies included was variable, including a mixture of patients with recurrent implantation failure, unexplained infertility and recurrent miscarriages, several dosing regimes, and co-treatment with other add-ons (37).

A more recent systematic review and meta-analysis of the available evidence regarding immunotherapy (38) identified 15 suitable RCTs, but only 2 of those were RCTs regarding IVIg in women undergoing IVF treatment with a history of RIF. These studies were already included in the previous meta-analysis by Li and colleagues 2013 (37); the number of patients was small, and pooling of their results did not demonstrate any difference between the study and the control group in terms of CPR or LBR (39,40). IVIg is a pooled blood product, and as such its use is not without risks. The majority of those are mild and transient; however, serious side effects, although rare, have been reported (41). Therefore, even without taking into consideration cost-effectiveness, their use cannot be recommended.

#### Anti TNF-α

An exaggerated Th1 response is thought to be detrimental to the process of embryo implantation and has been linked to infertility; therefore, elevated tumour necrosis factor (TNF) levels have been targeted for therapeutic correction in patients with a raised Th1/Th2 ratio. A potential reproductive benefit of anti-TNF-α agents, adalimumab (Humira), etanercept (Enbrel), and infliximab (Remicaid) has been proposed in infertile women with abnormal immunological parameters. A small retrospective case–control study of women with Th1/Th2 cytokine elevation reported a LBR of 73% in 41 patients who received IVIg and adalimumab, 50% in 6 patients who received adalimumab alone, and no live birth in 5 patients who received neither (42). Anti-TNF-α agents are also being used off-label for the treatment of endometriosis (43), and an interesting recent retrospective study suggested that etanercept may improve CPR but not LBR in women with an endometrioma having IVF (44). Any potential advantage from the use of anti-TNF-α has to be balanced against the known adverse effects of immune suppression such as the increased risk of lymphoma, skin cancer, and granulomatous infections. The risk profile of short-term use is less clear, but until well-designed, appropriately conducted large clinical trials clarify the role and safety of using anti-TNF-α agents as an adjuvant treatment to improve IVF outcome, their use cannot be supported.

#### Granulocyte colony-stimulating factor (G-CSF)

G-CSF is a cytokine that stimulates neutrophilic granulocyte proliferation and differentiation. It has been proposed as an important factor for embryo extravillous trophoblast cells invasion and adequate placentation. It has been used for patients with RIF but also for those with thin endometrium. Despite a rapidly increasing interest over recent years, the actions of G-CSF are still not clarified. Existing studies are very heterogeneous, include different regimes and routes of administration (intrauterine versus subcutaneous), and their interpretation should be carried out with caution. Two very recent attempts to systematically review results from these studies reported conflicting results. Zhang and colleagues reviewed 10 studies of various design and demonstrated improved CPR with the use of G-CSF in IVF cycles regardless of the route of administration for cases with RIF (45). On the other hand, Achilli and colleagues identified only five suitable studies for their review, which evaluated women undergoing IVF treatment with or without a history of RIF. Pooling of the results from two studies of the unselected IVF population demonstrated no difference in terms of CPR (OR = 0.99; 95% CI, 0.53, 1.84; P = .98). For the other three studies looking in the RIF population, the authors did not pool the results due to the different ways of administration (subcutaneous, intrauterine, or unspecified). One of those did not demonstrate any difference in CPR (intrauterine route), but the other two did show improvement with the use of G-CSF (38). Notably, a retrospective study did not report any adverse safety outcomes in the context of IVF by the use of G-CSF (46). Whilst this is reassuring, more studies are awaited to shed light on regimes, route of administration, and groups of patients that may benefit from this promising intervention.

### Dehydroepiandrosterone

Dehydroepiandrosterone (DHEA) is an endogenous steroid hormone produced primarily in the adrenal glands, gonads, and brain. It is difficult to distinguish it from its sulphated product, and it is known to decline with advancing chronological age. It is believed that it can enhance follicular function by increasing the production of insulin-like growth factor-1 (IGF-1) and augmenting oestradiol production in granulosa cells, acting as a precursor of androstenedione and testosterone in the ovarian theca cells. In addition, it has been suggested that pre-treatment with DHEA can reduce embryo aneuploidy, possibly by improving the ovarian micro-environment in which follicular maturation occurs (47).

A worldwide survey conducted in 2010 revealed that over one‐quarter of specialists add DHEA as an adjuvant to IVF treatment for anticipated low responders. A dosing regime of 25 mg three times daily is standard and has been used in most of the available studies which makes evidence less heterogeneous.

A plethora of publications on the positive effects of DHEA on ovarian response, embryo quality, and pregnancy outcomes in poor responders undergoing IVF are available. Nevertheless, there is still no consensus regarding its perceived benefit, and many of the available studies have been subsequently criticised by others. A large RCT investigating 12 weeks of DHEA supplementation in 104 poor responders compared with 104 controls found an insignificantly higher pregnancy rate in the control group (48).

Conversely, a Cochrane review revealed higher ongoing pregnancy rates and LBR following DHEA use; however, the benefit was not statistically significant (OR 1.50, 95% CI 0.88–2.56) when studies with high risk of performance bias were excluded (49).

The DITTO (Dehydroepiandrosterone Intervention to Treat Ovarian Aging) single-center RCT included a small number of subjects (25 versus 27 in each arm) and failed to demonstrate any benefit from the use of DHEA in women with diminished ovarian reserve undergoing IVF (50).

Other attempts for meta-analyses that followed failed to be conclusive. Qin and colleagues suggested significantly increased CPR for patients with diminished ovarian reserve who were pre-treated with DHEA when data from nine studies of various design were included. There was, however, no significant increase when the data were restricted to RCTs (51). A later meta-analysis including nine RCTs reported higher CPR, number of retrieved oocytes, and LBR in women who had been pre-treated with DHEA prior to IVF. However, the studies included had high risk of bias, and some used alternative dosage for DHEA (52).

It is interesting that for the anticipated normal responder population a well-designed double-blind RCT did not report any difference in number of oocytes or ovarian response after 12 weeks of DHEA (53). However, this study did not report on pregnancy outcomes.

The absence of significant side effects should not be considered as an argument to recommend routine use of DHEA. Nevertheless, it is difficult to stop patients self-medicating especially since DHEA is readily available online and over the counter. Counselling is essential for patients, and it seems that the use of DHEA will not stop unless any new solid evidence, which dictates otherwise, becomes available.

### Growth hormone

Growth hormone (GH) is a requisite of normal puberty, appears to have a role in ovarian function, and is proposed to have a modulatory action on the effect of FSH on granulosa cells by up-regulating the local synthesis of IGF-1. This in turn is thought to enhance the effect of gonadotrophin action at the level of both the granulosa and theca cells. Although the potential role of GH in ART has been recognised and studied for more than three decades, over the recent years a newfound interest has emerged, and GH has again become a popular topic. A Cochrane review published in 2010 included 10 RCTs with a total of 440 subjects (54). The authors observed considerable heterogeneity in the definitions, protocols, and outcomes. No benefit was seen by the use of GH supplementation in women considered as normal responders, whereas those considered to be poor responders had a significant benefit in both CPR (OR 3.28, 95% CI 1.74–6.20) and LBR (OR 5.39, 95% CI 1.89–15.35). The reviewers also put a caution on interpreting the result as the RCTs included in the meta-analyses were too few in number and too small in sample size to draw a definitive conclusion. The advantage of adding GH seemed to be limited to treatment cycles with GnRH agonist protocol only, and in a later study no significant benefit was found by adding GH in antagonist cycles (55).

In 2015, an updated meta-analysis reported results in line with previous evidence. The results showed a significant improvement in terms of mature oocytes retrieved and number of embryos available for transfer by GH supplementation in poor responders. However, there was no significant difference in CPR (56). In some of the more recent studies participants were classified as poor responders according to approved criteria. In the study by Dakhly and colleagues there was no significant difference in CPR in 240 low responders (classified using the Bologna criteria) randomised to GH or placebo and undergoing a long down-regulation protocol of ovarian stimulation (57).

The latest meta-analysis regarding the use of GH in controlled ovarian stimulation (COS) was published in 2017 (58). It included all previous articles as well as data from the LIGHT study which was performed by the same group. The LIGHT study was a multicenter, double-blind, placebo-controlled trial performed in 10 centres throughout Australia and New Zealand. The authors did not use Bologna or POSEIDON criteria for poor ovarian reserve. After 4 years of enrolment, the study was interrupted prematurely without reaching the desired numbers due to difficulties in recruitment. The number of patients reaching an oocyte retrieval was significantly higher in the GH group; however, no differences were reported in the LBR (59). The meta-analysis of 2017 unsurprisingly showed no evidence of an increased chance of live birth by the use of GH, although there was a significant reduction in the duration of stimulation required and a greater number of oocytes were collected than in the placebo arm. GH together with its receptor, GH-r, and related growth factors are also expressed in the human endometrium and may even have an indirect role in the function and maintenance of the corpus luteum. An additional potential benefit at the level of the uterus has therefore been suggested especially among women with recurrent implantation failure or thin endometrium. Thus, some of the beneficial effects of GH on assisted reproduction outcomes that have been suggested in the literature may, at least partially, be a result of the action of GH on endometrial receptivity (60).

### Endometrial injury

Although injury to the endometrium prior to embryo transfer is not strictly speaking an adjuvant to IVF, we have included it in our review as it is an intervention which has gained a lot of interest over the recent years. The theory behind it was that it may positively affect implantation by inducing the release of factors such as cytokines, interleukins, and growth factors in the endometrium. The most common and easily available procedure was via the use of a pipelle known as ‘endometrial scratching’. After many published studies a RCT was conclusive in showing that endometrial scratching does not confer any benefit to reproductive outcomes, including LBR (61). An alternative to endometrial scratching which is also commonly practiced by many, is the routine use of outpatient hysteroscopy prior to starting an IVF cycle, which may diagnose subtle pathology but will also act similarly as endometrial injury. A well conducted, adequately powered, multicenter RCT failed to demonstrate any improvement in LBR by the use of the intervention for women with recurrent IVF failure (62).

## Conclusions

There are obviously more interventions used in IVF cycles that could be described as adjuvants, but our review cannot exhaust them all. Platelet-rich plasm injection in the ovaries, mitochondrial replacement therapy (spindle transfer), melatonin, mitochondria DNA load measurement, among others, may prove to live up to the hype in the future.

For most interventions mentioned in this document (summarized in Table 1) lack of satisfactory evidence may be interpreted as evidence of lack of benefit. This argument from ignorance or ‘*argumentum ad ignorantiam*’ is a famous informal fallacy in logic.

**Table 1.** Two broad categories are discussed, adjuvants that are related to the IVF laboratory per se and involve gametes or embryos, and medical adjuvants which include patient-centered treatments or interventions.

Type of adjuvant	Evidence for efficacy
Laboratory-related adjuvants	
Embryo glue and adherence compounds	++
Assisted hatching	−
Artificial oocyte activation (AOA)	±
Patient-centered adjuvants	
Improvement of blood supply	+
Antioxidants	+
Immunotherapy	±
DHEA	+
Growth hormone	−
Endometrial injury	−

− no evidence; + limited evidence; ++ moderate evidence; ± inconclusive evidence.

But, before we reject any add-on shown not to work or we embrace others where evidence is favourable, we need to ascertain its effect in patients with a specific profile that might be modulated by the treatment under consideration. Novel diagnostic technologies are emerging that will enhance our understanding of the nature and determinants of reproductive functions. These technologies pave the way to a personalised approach in medicine and promise to help us identify specific patients who may benefit or be harmed by an intervention. Dismissing adjuvants altogether may pose the risk of keeping us stuck in the ‘one-size-fits-all’ clinical paradigm. Until then keeping an open mind and being honest and transparent with the patient is the best way forward.

## References

[1] Bontekoe S, Heineman MJ, Johnson N, Blake D Adherence compounds in embryo transfer media for assisted reproductive technologies. Cochrane Database Syst Rev 2014;CD007421. 24567053 10.1002/14651858.CD007421.pub3 PMC6953409

[2] Li D, Yang DL, An J, Jiao J, Zhou YM, Wu QJ, et al. Effect of assisted hatching on pregnancy outcomes: a systematic review and meta-analysis of randomized controlled trials. Sci Rep. 2016;6:31228. 27503701 10.1038/srep31228 PMC4977517

[3] Sfontouris IA, Nastri CO, Lima ML, Tahmasbpourmarzouni E, Raine-Fenning N, Martins WP Artificial oocyte activation to improve reproductive outcomes in women with previous fertilization failure: a systematic review and meta-analysis of RCTs. Hum Reprod. 2015;30:1831–41. 26082476 10.1093/humrep/dev136

[4] Potdar N, Gelbaya TA, Konje JC, Nardo LG Adjunct low-molecular-weight heparin to improve live birth rate after recurrent implantation failure: a systematic review and meta-analysis. Hum Reprod Update. 2013;19:674–84. 23912476 10.1093/humupd/dmt032

[5] Akhtar MA, Sur S, Raine-Fenning N, Jayaprakasan K, Thornton J, Quenby S, et al. Heparin for assisted reproduction: summary of a Cochrane review. Fertil Steril. 2015;103:33–4. 25282470 10.1016/j.fertnstert.2014.09.005

[6] Seshadri S, Sunkara SK, Khalaf Y, El-Toukhy T, Hamoda H Effect of heparin on the outcome of IVF treatment: a systematic review and meta-analysis. Reprod Biomed Online. 2012;25:572–84. 23069743 10.1016/j.rbmo.2012.08.007

[7] Siristatidis C, Dafopoulos K, Salamalekis G, Galazios G, Christoforidis N, Moustakarias T, et al. Administration of low-molecular-weight heparin in patients with two or more unsuccessful IVF/ICSI cycles: a multicenter cohort study. Gynecol Endocrinol. 2018;34:747–51. 29465258 10.1080/09513590.2018.1442426

[8] Fluhr H, Spratte J, Ehrhardt J, Steinmuller F, Licht P, Zygmunt M Heparin and low-molecular-weight heparins modulate the decidualization of human endometrial stromal cells. Fertil Steril. 2010;93:2581–7. 19962139 10.1016/j.fertnstert.2009.10.025

[9] Bolnick AD, Bolnick JM, Kohan-Ghadr HR, Kilburn BA, Pasalodos OJ, Singhal PK, et al. Enhancement of trophoblast differentiation and survival by low molecular weight heparin requires heparin-binding EGF-like growth factor. Hum Reprod. 2017;32:1218–29. 28402449 10.1093/humrep/dex069 PMC6075585

[10] Rubinstein M, Marazzi A, Polak de Fried E Low-dose aspirin treatment improves ovarian responsiveness, uterine and ovarian blood flow velocity, implantation, and pregnancy rates in patients undergoing in vitro fertilization: a prospective, randomized, double-blind placebo-controlled assay. Fertil Steril. 1999;71:825–9. 10231040 10.1016/s0015-0282(99)00088-6

[11] Gelbaya TA, Kyrgiou M, Li TC, Stern C, Nardo LG Low-dose aspirin for in vitro fertilization: a systematic review and meta-analysis. Hum Reprod Update. 2007;13:357–64. 17347160 10.1093/humupd/dmm005

[12] Siristatidis CS, Dodd SR, Drakeley AJ Aspirin for in vitro fertilisation. Cochrane Database Syst Rev 2011;CD004832. 21833951 10.1002/14651858.CD004832.pub3

[13] de Jong PG, Kaandorp S, Di Nisio M, Goddijn M, Middeldorp S Aspirin and/or heparin for women with unexplained recurrent miscarriage with or without inherited thrombophilia. Cochrane Database Syst Rev 2014;CD004734. 24995856 10.1002/14651858.CD004734.pub4 PMC6769058

[14] Sher G, Fisch JD Effect of vaginal sildenafil on the outcome of in vitro fertilization (IVF) after multiple IVF failures attributed to poor endometrial development. Fertil Steril. 2002;78:1073–6. 12413996 10.1016/s0015-0282(02)03375-7

[15] Check JH, Graziano V, Lee G, Nazari A, Choe JK, Dietterich C Neither sildenafil nor vaginal estradiol improves endometrial thickness in women with thin endometria after taking oral estradiol in graduating dosages. Clin Exp Obstet Gynecol 2004;31:99–102. 15266759

[16] Agarwal A, Parekh N, Panner Selvam MK, Henkel R, Shah R, Homa ST, et al. Male oxidative stress infertility (MOSI): proposed terminology and clinical practice guidelines for management of idiopathic male infertility. World J Mens Health. 2019;37:296–312. 31081299 10.5534/wjmh.190055 PMC6704307

[17] Showell M, Brown J, O’Donnell J, Hart R Antioxidants for female subfertility. The Cochrane. Database Syst Rev 2013;8:CD007807. 10.1002/14651858.CD007807.pub2 23913583

[18] Marron K, Kennedy JF, Harrity C Anti-oxidant mediated normalisation of raised intracellular cytokines in patients with reproductive failure. Fertil Res Pract 2018;4:1. 29507746 10.1186/s40738-018-0046-4 PMC5833037

[19] Ben-Meir A, Burstein E, Borrego-Alvarez A, Chong J, Wong E, Yavorska T, et al. Coenzyme Q10 restores oocyte mitochondrial function and fertility during reproductive aging. Aging Cell. 2015;14:887–95. 26111777 10.1111/acel.12368 PMC4568976

[20] Ben-Meir A, Kim K, McQuaid R, Esfandiari N, Bentov Y, Casper RF, et al. Co-enzyme Q10 supplementation rescues cumulus cells dysfunction in a maternal aging model. Antioxidants (Basel). 2019;8:58. 10.3390/antiox8030058 PMC6466589 30857157

[21] Xu Y, Nisenblat V, Lu C, Li R, Qiao J, Zhen X, et al. Pretreatment with coenzyme Q10 improves ovarian response and embryo quality in low-prognosis young women with decreased ovarian reserve: a randomized controlled trial. Reprod Biol Endocrinol. 2018;16:29. 29587861 10.1186/s12958-018-0343-0 PMC5870379

[22] Poljsak B, Šuput D, Milisav I Achieving the balance between ROS and antioxidants: when to use the synthetic antioxidants. Oxid Med Cell Longev. 2013;2013:1–11. 10.1155/2013/956792 PMC3657405 23738047

[23] Moffett A, Loke YW The immunological paradox of pregnancy: a reappraisal. Placenta 2004;25:1–8. 15013633 10.1016/S0143-4004(03)00167-X

[24] Moffett A, Shreeve N First do no harm: uterine natural killer (NK) cells in assisted reproduction. Hum Reprod. 2015;30:1519–25. 25954039 10.1093/humrep/dev098 PMC4472320

[25] Tang AW, Alfirevic Z, Quenby S Natural killer cells and pregnancy outcomes in women with recurrent miscarriage and infertility: a systematic review. Hum Reprod. 2011;26:1971–80. 21613313 10.1093/humrep/der164

[26] Boomsma CM, Macklon NS Does glucocorticoid therapy in the peri-implantation period have an impact on IVF outcomes? Curr Opin Obstet Gynecol. 2008;20:249–56. 18460939 10.1097/GCO.0b013e3282f8aff5

[27] Boomsma CM, Keay SD, Macklon NS Peri-implantation glucocorticoid administration for assisted reproductive technology cycles. Cochrane Database Syst Rev 2012;CD005996. 17253574 10.1002/14651858.CD005996.pub2

[28] Polanski LT, Barbosa MA, Martins WP, Baumgarten MN, Campbell B, Brosens J, et al. Interventions to improve reproductive outcomes in women with elevated natural killer cells undergoing assisted reproduction techniques: a systematic review of literature. Hum Reprod. 2014;29:65–75. 24256994 10.1093/humrep/det414

[29] Dan S, Wei W, Yichao S, Hongbo C, Shenmin Y, Jiaxiong W, et al. Effect of prednisolone administration on patients with unexplained recurrent miscarriage and in routine intracytoplasmic sperm injection: a meta-analysis. Am J Reprod Immunol. 2015;74:89–97. 25753479 10.1111/aji.12373

[30] Siristatidis C, Chrelias C, Creatsa M, Varounis C, Vrachnis N, Iliodromiti Z, et al. Addition of prednisolone and heparin in patients with failed IVF/ICSI cycles: a preliminary report of a clinical trial. Hum Fertil (Camb). 2013;16:207–10. 23834353 10.3109/14647273.2013.803608

[31] Fawzy M, El-Refaeey AA Does combined prednisolone and low molecular weight heparin have a role in unexplained implantation failure? Arch Gynecol Obstet. 2014;289:677–80. 24048369 10.1007/s00404-013-3020-8

[32] Park-Wyllie L, Mazzotta P, Pastuszak A, Moretti ME, Beique L, Hunnisett L, et al. Birth defects after maternal exposure to corticosteroids: prospective cohort study and meta-analysis of epidemiological studies. Teratology 2000;62:385–92. 11091360 10.1002/1096-9926(200012)62:6<385::AID-TERA5>3.0.CO;2-Z

[33] Roussev RG, Acacio B, Ng SC, Coulam CB Duration of intralipid’s suppressive effect on NK cell’s functional activity. Am J Reprod Immunol. 2008;60:258–63. 18782287 10.1111/j.1600-0897.2008.00621.x

[34] Dakhly DM, Bayoumi YA, Sharkawy M, Gad Allah SH, Hassan MA, Gouda HM, et al. Intralipid supplementation in women with recurrent spontaneous abortion and elevated levels of natural killer cells. Int J Gynaecol Obstet. 2016;135:324–7. 27614789 10.1016/j.ijgo.2016.06.026

[35] Check JH, Check DL Intravenous intralipid therapy is not beneficial in having a live delivery in women aged 40-42 years with a previous history of miscarriage or failure to conceive despite embryo transfer undergoing in vitro fertilization-embryo transfer. Clin Exp Obstet Gynecol 2016;43:14–5. 27048011

[36] Hirshfeld-Cytron JE, Martini A, Jasulaitis S, Uhler ML Does intralipid infusion improve outcomes in RPL (recurrent pregnancy loss)/RIF (recurrent implantation failure) patients undergoing IVF? is it cost-effective?. Fertil Steril. 2016;106:e342.

[37] Li J, Chen Y, Liu C, Hu Y, Li L Intravenous immunoglobulin treatment for repeated IVF/ICSI failure and unexplained infertility: a systematic review and a meta-analysis. Am J Reprod Immunol. 2013;70:434–47. 24238107 10.1111/aji.12170

[38] Achilli C, Duran-Retamal M, Saab W, Serhal P, Seshadri S The role of immunotherapy in in vitro fertilization and recurrent pregnancy loss: a systematic review and meta-analysis. Fertil Steril. 2018;110:1089–100. 30396553 10.1016/j.fertnstert.2018.07.004

[39] De Placido G, Zullo F, Mollo A, Cappiello F, Nazzaro A, Colacurci N, et al. Intravenous immunoglobulin (IVIG) in the prevention of implantation failures. Ann NY Acad Sci. 1994;734:232–4. 7978921 10.1111/j.1749-6632.1994.tb21751.x

[40] Stephenson MD, Fluker MR Treatment of repeated unexplained in vitro fertilization failure with intravenous immunoglobulin: a randomized, placebo-controlled Canadian trial. Fertil Steril. 2000;74:1108–13. 11119735 10.1016/s0015-0282(00)01622-8

[41] Souayah N, Hasan A, Khan HM, Yacoub HA, Jafri M The safety profile of home infusion of intravenous immunoglobulin in patients with neuroimmunologic disorders. J Clin Neuromuscul Dis 2011;12:S1–S10. 22361589 10.1097/CND.0b013e3182212589

[42] Winger EE, Reed JL, Ashoush S, Ahuja S, El-Toukhy T, Taranissi M Treatment with adalimumab (Humira) and intravenous immunoglobulin improves pregnancy rates in women undergoing IVF. Am J Reprod Immunol. 2009;61:113–20. 19055656 10.1111/j.1600-0897.2008.00669.x

[43] Quaas AM, Weedin EA, Hansen KR On-label and off-label drug use in the treatment of endometriosis. Fertil Steril. 2015;103:612–25. 25637479 10.1016/j.fertnstert.2015.01.006

[44] Önalan G, Tohma YA, Zeyneloğlu HB Effect of etanercept on the success of assisted reproductive technology in patients with endometrioma. Gynecol Obstet Invest. 2018;83:358–64. 29208847 10.1159/000484895

[45] Zhang L, Xu W-H, Fu X-H, Huang Q-X, Guo X-Y, Zhang L, et al. Therapeutic role of granulocyte colony-stimulating factor (G-CSF) for infertile women under in vitro fertilization and embryo transfer (IVF-ET) treatment: a meta-analysis. Arch Gynecol Obstet. 2018;298:861–71. 30220024 10.1007/s00404-018-4892-4 PMC6182707

[46] Cruz M, Alecsandru D, Garcia-Velasco JA, Requena A Use of granulocyte colony-stimulating factor in ART treatment does not increase the risk of adverse perinatal outcomes. Reprod Biomed Online. 2019;39:976–80. 31680063 10.1016/j.rbmo.2019.09.008

[47] Gleicher N, Weghofer A, Barad DH Dehydroepiandrosterone (DHEA) reduces embryo aneuploidy: direct evidence from preimplantation genetic screening (PGS). Reprod Biol Endocrinol. 2010;8:140. 21067609 10.1186/1477-7827-8-140 PMC2992540

[48] Kara M, Aydin T, Aran T, Turktekin N, Ozdemir B Does dehydroepiandrosterone supplementation really affect IVF-ICSI outcome in women with poor ovarian reserve?. Eur J Obstet Gynecol Reprod Biol. 2014;173:63–5. 24331115 10.1016/j.ejogrb.2013.11.008

[49] Nagels HE, Rishworth JR, Siristatidis CS, Kroon B Androgens (dehydroepiandrosterone or testosterone) for women undergoing assisted reproduction. Cochrane Database Syst Rev 2015;CD009749. 26608695 10.1002/14651858.CD009749.pub2 PMC10559340

[50] Narkwichean A, Maalouf W, Baumgarten M, Polanski L, Raine-Fenning N, Campbell B, et al. Efficacy of dehydroepiandrosterone (DHEA) to overcome the effect of ovarian ageing (DITTO): a proof of principle double blinded randomized placebo controlled trial. Eur J Obstet Gynecol Reprod Biol. 2017;218:39–48. 28934714 10.1016/j.ejogrb.2017.09.006

[51] Qin JC, Fan L, Qin AP The effect of dehydroepiandrosterone (DHEA) supplementation on women with diminished ovarian reserve (DOR) in IVF cycle: evidence from a meta-analysis. J Gynecol Obstet Hum Reprod. 2017;46:1–7. 28403950 10.1016/j.jgyn.2016.01.002

[52] Xu L, Hu C, Liu Q, Li Y The effect of dehydroepiandrosterone (DHEA) supplementation on IVF or ICSI: a meta-analysis of randomized controlled trials. Geburtshilfe Frauenheilkd. 2019;79:705–12. 31303658 10.1055/a-0882-3791 PMC6620181

[53] Yeung T, Chai J, Li R, Lee V, Ho PC, Ng E A double-blind randomised controlled trial on the effect of dehydroepiandrosterone on ovarian reserve markers, ovarian response and number of oocytes in anticipated normal ovarian responders. BJOG: Int J Obstet Gy. 2016;123:1097–105. 10.1111/1471-0528.13808 26663817

[54] Duffy JM, Ahmad G, Mohiyiddeen L, Nardo LG, Watson A Growth hormone for in vitro fertilization. Cochrane Database Syst Rev 2010;CD000099. 20091500 10.1002/14651858.CD000099.pub3 PMC7058116

[55] Eftekhar M, Aflatoonian A, Mohammadian F, Eftekhar T Adjuvant growth hormone therapy in antagonist protocol in poor responders undergoing assisted reproductive technology. Arch Gynecol Obstet. 2013;287:1017–21. 23208461 10.1007/s00404-012-2655-1

[56] Yu X, Ruan J, He LP, Hu W, Xu Q, Tang J, et al. Efficacy of growth hormone supplementation with gonadotrophins in vitro fertilization for poor ovarian responders: an updated meta-analysis. Int J Clin Exp Med 2015;8:4954–67. 26131068 PMC4483949

[57] Dakhly DMR, Bassiouny YA, Bayoumi YA, Hassan MA, Gouda HM, Hassan AA The addition of growth hormone adjuvant therapy to the long down regulation protocol in poor responders undergoing in vitro fertilization: Randomized control trial. Eur J Obstet Gynecol Reprod Biol. 2018;228:161–5. 29957401 10.1016/j.ejogrb.2018.06.035

[58] Hart RJ, Rombauts L, Norman RJ Growth hormone in IVF cycles: any hope?. Curr Opin Obstet Gynecol. 2017;29:119–25. 28306560 10.1097/GCO.0000000000000360

[59] Norman RJ, Alvino H, Hull LM, Mol BW, Hart RJ, Kelly TL, et al. Human growth hormone for poor responders: a randomized placebo-controlled trial provides no evidence for improved live birth rate. Reprod Biomed Online. 2019;38:908–15. 30954433 10.1016/j.rbmo.2019.02.003

[60] Altmae S, Aghajanova L Growth hormone and endometrial receptivity. Front Endocrinol (Lausanne) 2019;10:653. 31616379 10.3389/fendo.2019.00653 PMC6768942

[61] Lensen S, Osavlyuk D, Armstrong S, Stadelmann C, Hennes A, Napier E, et al. A randomized trial of endometrial scratching before in vitro fertilization. N Engl J Med. 2019;380:325–34. 30673547 10.1056/NEJMoa1808737

[62] El-Toukhy T, Campo R, Khalaf Y, Tabanelli C, Gianaroli L, Gordts SS, et al. Hysteroscopy in recurrent in-vitro fertilisation failure (TROPHY): a multicentre, randomised controlled trial. Lancet 2016;387:2614–21. 27132053 10.1016/S0140-6736(16)00258-0

